# Metabolic Profile of the Cellulolytic Industrial Actinomycete *Thermobifida fusca*

**DOI:** 10.3390/metabo7040057

**Published:** 2017-11-11

**Authors:** Niti Vanee, J. Paul Brooks, Stephen S. Fong

**Affiliations:** 1 VCU Life Sciences, Virginia Commonwealth University, Richmond 23284, VA, USA; nitivanee@gmail.com (N.V.); jpbrooks@vcu.edu (J.P.B.); 2 Chemical and Life Sciences Engineering, Virginia Commonwealth University, Richmond 23284, VA, USA

**Keywords:** metabolomics, metabolites, metabolic model, actinomycetes, cellobiose, avicel

## Abstract

Actinomycetes have a long history of being the source of numerous valuable natural products and medicinals. To expedite product discovery and optimization of biochemical production, high-throughput technologies can now be used to screen the library of compounds present (or produced) at a given time in an organism. This not only facilitates chemical product screening, but also provides a comprehensive methodology to the study cellular metabolic networks to inform cellular engineering. Here, we present some of the first metabolomic data of the industrial cellulolytic actinomycete *Thermobifida fusca* generated using LC-MS/MS. The underlying objective of conducting global metabolite profiling was to gain better insight on the innate capabilities of *T. fusca*, with a long-term goal of facilitating *T. fusca*-based bioprocesses. The *T. fusca* metabolome was characterized for growth on two cellulose-relevant carbon sources, cellobiose and Avicel. Furthermore, the comprehensive list of measured metabolites was computationally integrated into a metabolic model of *T. fusca*, to study metabolic shifts in the network flux associated with carbohydrate and amino acid metabolism.

## 1. Introduction

High-throughput experimental data (genomics, transcriptomics, proteomics, metabolomics) provide distinct glimpses into cellular function. When considering cellular metabolism, metabolomics is unique in being a direct outcome measure of integrated biochemical function, and can provide the starting point information for efficient pathway engineering. However, the dynamics of metabolomic data is not only defined by genetic and environmental factors, but also the life stages of the bacteria. As a broad classification, the difference between the primary and secondary metabolites is the growth phase when it is produced in the bacteria. The early log phase shows a higher concentration of primary metabolites, which is utilized for growth, and reproduction of the cells. This is followed by the production of secondary metabolites as the byproducts of the metabolic process. Many of these secondary metabolites are known for their medical and industrial relevance.

Actinomycetes have a long-cited history for producing medically-relevant chemicals, beginning with antibiotics (roughly two-thirds of clinically used antibiotics are produced by the actinomycete, *Streptomyces*). As actinomycetes continue to be studied, additional important compounds, such as siderophores [1], polyketides [2,3], and terpenoids [4,5], continue to be identified. The breadth of compounds natively produced by actinomycetes suggests that this group of Gram-positive bacteria may be naturally well-suited for production of medically-relevant compounds. This is supported by the fact that approximately 45 percent of all bioactive compounds obtained from microorganisms come from actinomycetes [4].

*Thermobifida fusca* is a soil bacteria that belongs to the family of actinomycetes. In this study, our goal is to gain a better understanding of the metabolic capabilities of *T. fusca* by measuring the *T. fusca* metabolome, and integrating this data with a computational metabolic model. This study is meant to extend previous work where metabolism in *T. fusca* was characterized using systems-level modeling based on genomic and proteomic data [6]. Specifically, the macromolecular network of *T. fusca* is studied using metabolite profiling for growth on two different media formulations (cellobiose and Avicel). Cellobiose has been studied in past as the inducer of cellulases [7,8,9,10,11,12,13], thus, is used as the reference media condition. Avicel, on the other hand, is a crystalline solid carbon source more closely related to plant waste, and therefore, used for comparative analysis.

## 2. Results

Metabolomic data was obtained for growth on cellobiose or Avicel with separate samples being analyzed for detection of polar and non-polar metabolites for each growth condition.

### 2.1. Coverage Polar and Non-Polar Metabolites

All the samples were run with technical replicates, and the metabolites detected in both the replicates were used for further processing and analysis. For growth of the wild type *T. fusca* strain on cellobiose, 618 polar and 64 non-polar compounds were detected. For growth on Avicel, 402 polar and 82 non-polar compounds were detected. Of the detected compounds, 15 (cellobiose) and 23 (Avicel) were detected in both polar and non-polar runs. A summary of the number of detected compounds is shown in Table 1.

### 2.2. Functional Association of Metabolites

The MBRole [14] software was used for identifying functional associations of the detected metabolites. MBRole provides access to organism-specific precompiled lists of metabolites on KEGG as a reference [15,16]. The mapping category includes metabolic pathways, enzyme interactions, biological roles, chemical groups, and other interactions (transport associated molecules). The summary of functional classifications for detected metabolites is shown in Table 2.

### 2.3. Metabolites for Cellobiose Growth

Out of 667 metabolites detected in the cellobiose growth condition, no annotation could be detected for 474 compounds in *T. fusca*. For the detected compounds with annotations, the following were classified into the following categories: sugars (10 metabolites), lipids or fatty acids associated molecules (36), nucleotides (48), peptides or amino acids molecules (40), hormone or neurotransmitter like molecules (2), and vitamin molecules (9), which were detected in cellobiose growth condition. Eighty-one metabolites from secondary metabolism (or phytochemical compound) as named by KEGG were also identified. The other interactions associated with transporters (47), reporters (61), and ion channel-associated molecules were also identified. The transporter molecules included ABC type, and amino acid transporters (arginine, glycine, proline, methionine, arginine/ornithine, and glutamate). Among the receptor molecules, 24 glutamate receptors, including metabotropic and ionotropic subclass, were identified. The ionotropic receptors are the ligand gated non-selective ion channels and metabotropic activates biochemical cascades [17]. As compared to eukaryotic homologs, the prokaryotic glutamate receptors are less well-characterized. The detection of 24 compounds from this class opens an arena for further explorations of this family in *T. fusca.* This might also be useful to explain the strong secretome as studied in past [18].

Compounds associated with cytochrome P450 were another important family of compounds that was observed in the analysis. There were 21 metabolites associated with the CYP1 and CPY2 families. The primary function of this superfamily of proteins is to catalyze the oxidation of lipids, steroids and xenobiotic compounds, such as toxic chemicals and drugs [19].

Apart from learning the biological role of the molecules, these detected compounds were also mapped to the metabolic capabilities as pre-compiled by KEGG, using the open access online database interface MBRole [14]. Among these 206 detected compounds with annotation, 105 were associated with primary metabolism pathways, and 54 belonged to the secondary metabolism network. These networks are shown in Supplementary Figure S1a,b with compounds highlighted with grey. The major metabolic subsystems with the highest representation of detected metabolites were 38% in oxidative phosphorylation (tfu00190), with 38% of associated metabolites detected and nucleotide metabolism with 23% associated metabolites detected. For nucleotide metabolism, pyrimidine metabolism had 14 metabolites detected and purine metabolism had 21 metabolites detected. Detected metabolites associated with amino acids, carbohydrate, and secondary metabolism pathways are shown in Supplementary Table S1.

### 2.4. Metabolites for Avicel Growth

For growth of wild type *T. fusca* on Avicel, 461 compounds were detected, but 330 metabolites could not be associated with a functional category in *T. fusca* as defined by KEGG. The macromolecular distribution of annotated metabolites for growth on Avicel was: 7 sugar molecules, 21 lipids, 17 nucleotides, 43 peptides (and amino acids), and 6 vitamins. Most of the numbers were lesser as compared to cellobiose condition analysis, except for the number of identified peptides. For the transporter and receptor-associated compounds, the numbers were 43 and 41, respectively. There were 17 cytochrome P450 subfamily proteins and 19 glutamate receptor molecules (ionotropic and metabotropic) identified. One unique entity detected in the Avicel condition was 2-aminoethylphosphonate transporters, which are an ABC-type transporter that helps transport 2-aminoethylphosphonate across the membrane for utilization in bacteria. Bacteria has the capability to use these organophosphates as a source of carbon, energy, and phosphorous for growth. These organophosphates are often found in insecticides and herbicides [20].

Apart from the macromolecular classification, 131 identified compounds were studied for connections to metabolic pathways. Seventy-one metabolites were found to be associated with primary metabolic process, and 31 with secondary metabolism pathway. These networks are shown in Supplementary Figure S2a,b, with detected metabolites highlighted with grey. The metabolic subsystem with the highest percent of experimentally detected metabolites was lysine biosynthesis (tfu00300), where 9 out of 32 compounds (28%) were identified experimentally. Other detected compounds from carbohydrate, amino acid, and secondary metabolism pathways are listed in Supplementary Table S2.

### 2.5. Comparing Cellobiose vs Avice

**Figure 1:**
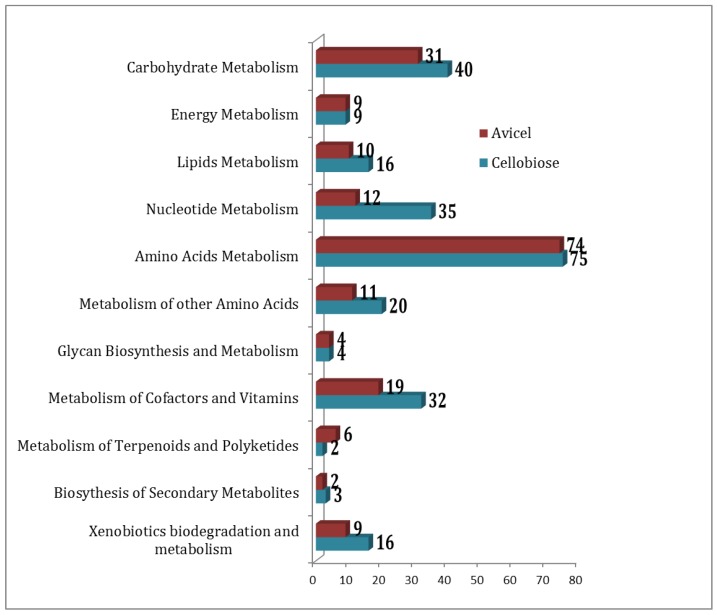
Distribution of compounds under the metabolic pathways as detected in cellobiose (blue) and Avicel (red) media condition formulation.

Overall, 827 unique metabolites were detected when combining results for growth on cellobiose and on Avicel. Three hundred and one metabolites were common to both growth conditions, 366 were unique to cellobiose, and 160 were unique to the Avicel growth condition. The pathway association of the compounds in these two conditions has been summarized in Figure 1.

When MBRole maps a metabolite to a metabolic pathway, it assigns a score of significance based on the background compounds known for each pathway versus the mapped list. Table 3 summarizes the significantly different pathway along with their uniquely detected compounds.

Some of the interesting compounds, besides the above list, that were unique to cellobiose media conditions, were dihydrostreptomycin 6-phosphate (C01221) from the streptomycin biosynthesis pathway, hexadecanoic acid (C00249) of fatty acid metabolism, lipoyl-AMP (C16238) of lipoic acid and D-alanine (C00041).

Among the 301 compounds whose peaks were detected in both cellobiose and Avicel condition, 88 were observed in differential quantities. Nineteen (out of 88) were detected at higher concentration in cellobiose, and 36 (out of 88) were higher in Avicel. These compounds when mapped with biological functions that belonged to fatty acid synthesis (C05763); lysine biosynthesis (C03871, C12986); arginine and proline biosynthesis (C00624, C03415), and glycerophospholipid metabolism (C00513) for the cellobiose condition. In the Avicel growth conditions, ubiquinone and other terpenoid-quinone biosynthesis (C00811); lysine degradation (C03955); phenylalanine metabolism (C00811); gamma-hexachlorocyclohexane degradation (C12836) and selenoamino acid metabolism (C05699) were the pathways for which the respective compounds were detected in higher concentrations. A summarized list of compounds is shown in Table 4.

### 2.6. Metabolites in Secondary Metabolism

The global metabolite detection was done for the general metabolites and mapped to the metabolic pathway of *T. fusca* using MBRole. As per the KEGG’s compilation of tfu01110 Biosynthesis of Secondary Metabolites, there were a total of 1038 compounds in the list. This compilation includes intermediate metabolites and the final products. The metabolomics data for growth on cellobiose and growth on Avicel was compared to the list of 1038 secondary metabolite compounds, and 63 metabolites associated with secondary metabolism were identified. To find of the missing nodes of the pre-compiled tfu01110 list, the metabolomics data was mapped to the list of all the natural products (total 2480). This gave an interesting list of 88 secondary metabolites. This breaks down into a total of alkaloids (41), terpenoids (25), phenylpropanoids (8), amino acid-related compounds (8), polyketides (4), flavonoids (4), and fatty acids related compounds (2). The details of these subcategories can be seen in Table 5. The list of compounds can be obtained in Supplementary Table S1 for growth on cellobiose, Supplementary Table S2 for growth on Avicel, and raw data in Supplementary Table S3.

One of the interesting findings from focusing on secondary metabolism was identification of the hemiterpenoid, isoprene. Past studies have shown that both Gram-positive and Gram-negative bacteria possess the capability to produce isoprene [21]. A comparative study between *Bacillus* sp., *Micrococcus*, *Rhodococcus*, *E. coli*, *Pseudomonas*, and *Agrobacterium* was conducted, and concluded that *Bacillus* ranks highest among all in the production of isoprene. The study illustrated the capability of Gram-positive bacteria to produce this industrially significant product. In our metabolomics analysis for *T. fusca*, we propose that this actinomycete also possesses similar characteristics and the ability to produce isoprene. The biological role of this compound as defined by Sharkey et al. in 2007 [22] as protection against the temperature stress (~40 °C). This might be useful to understand the mechanism of thermostability in *T. fusca*, a naturally thermophilic organism.

### 2.7. Integrating Metabolomic Data with a Metabolic Model

The predictive and analytical capabilities of constraint-based metabolic models can be significantly improved by integrating experimental data as model constraints. To gain a better in-context understanding of the metabolomics data, and to further improve our previously published metabolic model of *T. fusca* [6], we implemented a method for directly integrating metabolomics data into a constraint-based model.

For this study, we have focused only on data associated with growth on cellobiose, since the published *T. fusca* model was also based on growth on cellobiose. Starting with 946 reactions in the proteomics-based metabolic model of *T. fusca*, flux balance analysis predicted 56 reactions with low flux and 173 reactions with high flux, i.e., a total of 229 active flux reactions. Metabolomic data from this study was integrated into the metabolic model using the metabolites with high-confidence annotations, and the pathways with active fluxes was reduced to 224 (57 low flux reactions and 167 high flux reactions).

The reaction distribution in FBA model (without metabolomics data) and FBA with metabolomics data from cellobiose media is discussed in the following three categories: (1) reactions that remain unchanged in both scenarios (with and without metabolomics data integration), (2) reactions that were active in both scenarios, but there was a change in the flux value, and (3) reactions that were inactive in one scenario, but were active in the other.

**Unchanged reactions**: There were 773 out of 946 reactions that remain unchanged. Accounting for reactions with the same active flux in both scenarios, we found 60 reactions had same active flux. This involves 20 amino acid pathway reactions, 12 carbohydrate metabolism pathways, and 9 nucleotide metabolism pathways. These core, active pathways may constitute core metabolic pathways that remain constant, irrespective of the external parameters.

**Changed active reactions**: The reaction flux comparison with respect to FBA simulations (without metabolites data) can be divided into 4 parts—reactions with high flux (1000 to 250), moderate flux (249 to 3.8), low flux (3.8 to 0), and negative flux (0 to −1000).

**Figure 2:**
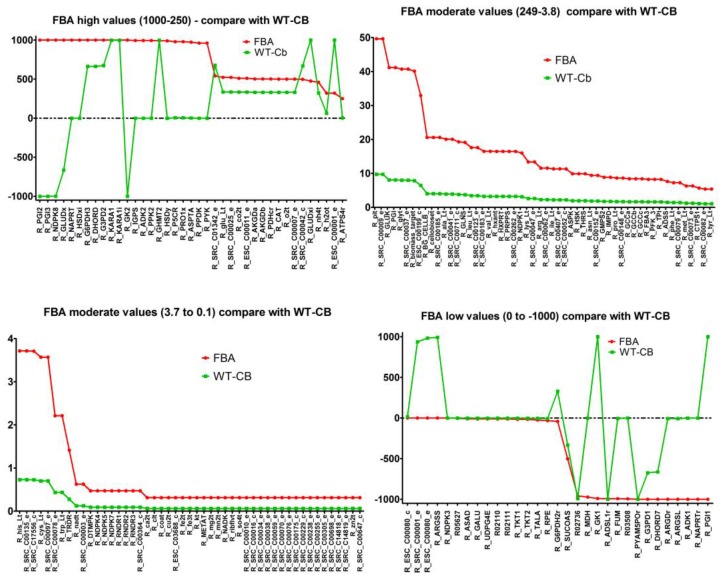
Comparison of metabolic pathways with high, medium, low, and negative fluxes between FBA simulations (red) and metabolomics-integrated simulations (green).

The overview of reaction flux variation can be seen in Figure 2 below.

It was observed that the moderate and low flux reactions showed an overall shift in the value to the lower range, however, the trend of which reactions were used remained largely unchanged. However, among the 39 reactions with high flux, there was a wide range of variability on how the flux changed for when the metabolites data was integrated.

**Figure 3:**
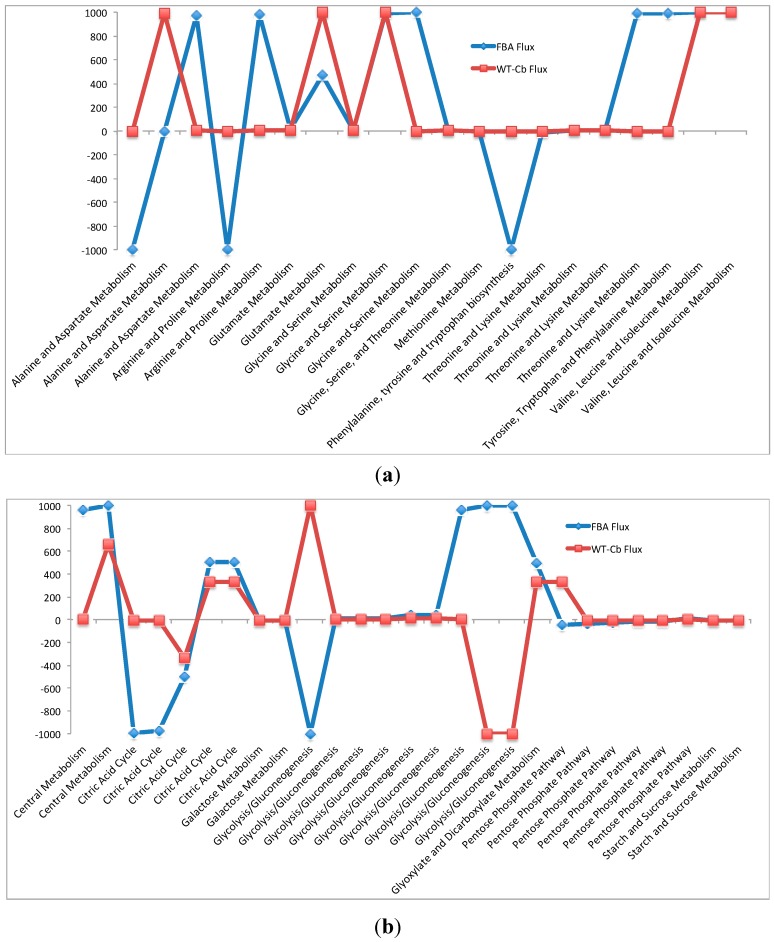
(**a**) Variation in Amino Acid Metabolism—with and without metabolomics data integration. (**b**)Variation in Carbohydrate Metabolism—with and without metabolomics data integration.

This variability is further explained with respect to amino acid (Figure 3a) and carbohydrate metabolism (Figure 3b). On integrating the metabolomic data, reactions between the arginine to fumarate changed from low flux to high flux. Also, the interconversion of arginine to citrulline was changed from low flux to moderate flux. Some of the major changes also include the inactivity around previously active reactions. These include reactions in tryptophan metabolism from chorismate and formation of lysine from homoserine. A significant drop in flux at formation of aspartate from oxaloacetate and formation of proline from arginine were also found.

In carbohydrate metabolism, significant changes were observed in glycolysis, TCA cycle, and pentose phosphate metabolism. In glycolysis, the pyruvate kinase mediated conversion of glucose to glucose 6-phosphate is reduced from a high flux reaction to inactive after data integration. In the TCA cycle, there is a positive shift (low flux to moderate flux) in all the reactions from succinyl-CoA to oxaloacetate, whereas there is a slight reduction in flux through intermediate reactions leading to formation of succinyl-CoA from alpha-ketoglutarate. In the pentose phosphate metabolism pathway, there is a significant increase in flux for the conversion of glucose 6-P to 6-phosphgluconolactone. This variability is illustrated in the graphs shown in Figure 3b. The detailed reactions list is presented in Table 6b, along with their fluxes values.

**Inactive to active change**: Finally, there was a set of nine reactions where flux balance analysis predicted active fluxes, but integration of metabolomic data indicated the reaction should be inactive (Table 7). Generally, the reactions that were found to be inactive after integration of metabolomics data were pathways where fluxes were easily routed to alternate pathways without significant effects on cellular phenotype.

## 3. Discussion

Metabolomics data provides direct evidence for the presence of substrate or product for all the cellular and biochemical reaction catalyzed in the cell at a specific time point. This data type has some unique strengths and weaknesses when used to study and analyze an organism’s metabolic capabilities and functionality. The global metabolomics profile of *T. fusca* presented here are the first ever metabolomic datasets, which can be used for the study of specific target molecules depending upon the application of pathway of focus.

One of the main strengths of metabolomics data is the direct detection of specific chemical species which is particularly useful when considering secondary metabolism. Due to the nuances and potential for unique secondary metabolic capabilities of an organism (which can vary much more than central metabolism), the biochemical production capabilities of a poorly-characterized organism are often speculative, if based upon genome-centric knowledge. In considering *T. fusca* as a potential cellolytic platform for producing biochemical products, there are a number of compounds that bioinformatically appear to have the potential to be natively produced, but have yet to be confirmed experimentally. In this study, we were able to identify several secondary metabolites produced by *T. fusca*, including isoprene, a high-value precursor metabolite that has not been previously identified for production in *T. fusca*.

An extended use of metabolomic data can be integration into other computational analysis approaches, such as metabolic models to help better understand the coordinated functionality of an organism’s metabolic network. In this study, we employed an algorithmic approach to integrate metabolomics data with a constraint-based metabolic model, focusing on using metabolites that were uniquely identified with high-confidence. Use of metabolomics data provides a completely complimentary type of information to the information that can be obtained from genomic, transcriptomic, or proteomic data. All of these data types can identify potential biochemical functionality, but they all also have the potential pitfall of being multifunctional (annotations for different functions for the same gene/protein). While genomic, transcriptomic, or proteomic data potentially has a wider coverage across a metabolic network, only metabolomics data has the potential to directly distinguish and confirm biochemical functionality. This helped confirm activity of some central metabolic pathways, and helped identify shifts in pathway usage.

In this study, the breadth of metabolomic data coverage across the metabolic network of *T. fusca* was limited, but the data that was obtained was used to help identify some differences in functional states between growth on cellobiose and Avicel. Most of the observed changes were consistent with what might be intuitively expected. Cellular growth is faster on cellobiose than on Avicel, and this is consistent with some of the changes that show increased fluxes and metabolites detected in amino acid synthesis that are required to support growth. Growth on Avicel is slower and requires secretion of proteins (e.g., cellulases) and uptake of a broader range of substrates, and thus, there was an increase in support subsystems and transporters. Furthermore, integration of metabolomics data with metabolic modeling was able to identify subtle shifts in pathway usage that were not possible using proteomic data.

The extent of utility of the metabolomics data in this study was severely limited by the ability to uniquely and definitively associate detected metabolites with a single chemical species. Raw metabolomic data currently suffers from a limitation of compound identification and accurate prediction due to the data available on the resource banks, such as KEGG, HMDB, etc. This is seen in compounds that have closely related masses and retention times. Some of the very closely related masses with similar retention times that were observed in the dataset are as follows: UDP (C00015), with the mass of 404.002200, and aluminoparaaminosalicylate calcium (C13104), with the mass of 404.011300. These two compounds vary at the second decimal place for the masses, and had similar retention time. Another example was 1-methyl-4-phenylpyridinium (C11310, mass = 170.097000) and furfural diethyl acetal (C14280, mass = 170.094300). Their mass varies at the third decimal place. Similarly, there were compounds that varied at fourth and fifth decimal points also having similar retention time. In our opinion, this is not the limitation of the detection technique, but more of the inadequacy at the resource bank end or the mapping/identification algorithm end. This leads to problems at both ends of the analysis pipeline, not only overestimation, but also sometimes underestimation or no annotations (in this case). Approximately, seventy percent of the compounds could not be well annotated in the context of the *T. fusca* pre-compiled compounds listed by KEGG. This was partially explained by limited functional annotation for *T. fusca* on KEGG. There was a significant increase in mapped compounds to this database. Due to these shortcomings, it is almost impossible to obtain an exhaustive coverage like in the case of genomics or transcriptomics level dataset. And if it is possible, the hierarchy of techniques as shown in Figure 1 will have to be followed to get the most comprehensive, confirmative, and accurate list of metabolites.

Thus, this method is useful to get an idea of focus targets, and has to be paired with the targeted metabolite search for the specific pathway of interest.

## 4. Materials and Methods

**Figure 4:**
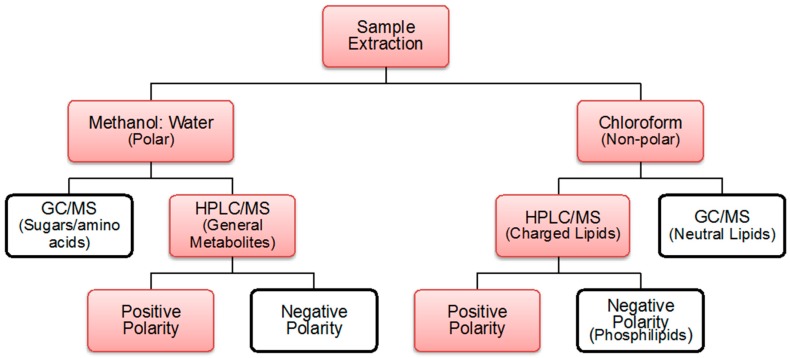
Metabolite profiling techniques used for the current study are highlighted in red. For both polar and non-polar metabolites, more exhaustive detections are observed in positive polarity mode, and thus, was the mode of choice in this experiment [23,24].

Global metabolite profiling experiments attempting to characterize all metabolites of a cellular system involve a huge combinatorial hierarchy of techniques [23]. Figure 4 shows the compound and technique classification that may be used for targeted or untargeted metabolite profiling. If a well-structured study is performed to attain an exhaustive metabolite profile of the microbe, it is possible to perform a top-down approach to forward engineer of a synthetic microbe with de novo synthesized genetic information to reach designed targeted production. This may further be developed as the designing principle for new synthetic microbes with most efficient manufacturing genetic and proteomic system.

In the current scope, only untargeted metabolite profiling using HPLC/MS-TOF in positive polarity was conducted. Using a methanol/water/chloroform extraction method, the aqueous and non-aqueous layers are separated for two different runs: polar and non-polar. The peak and mass data acquired on Agilent Mass Hunter Software from LC-MS was sent to the Omics Discovery Pipeline at Bindley Bioscience Center for deconvolution, alignment, normalization, and identification of the compounds [24]. The parameters for each step were established after observing the results from each previous step.

This analysis detects a subset of polar and non-polar compounds representing general metabolites and lipids, respectively. Technical replicates of *T. fusca* wild type grown on cellobiose and Avicel. This is the first ever attempt to investigate metabolomics of *T. fusca*. It opens an arena for target based metabolite profiling if required to specifically look into the pathways/product of interest. Raw metabolomics data is included in Supplementary Table S3.

### 4.1. Cell Growth

*T. fusca* YX strain (ATCC BAA-629) cells were grown on Hagerdahl media (ATCC medium: 2382) with cellobiose and Avicel as the carbon source (5.0 g/L) at 55 °C. The two growth conditions resulted in the dry cell weight of 8.4 mg/mL for cellobiose, and 4.5 mg/mL for growth on Avicel. Analysis and preparation of cells for metabolomics analysis was conducted during exponential growth.

### 4.2. Extraction Process

Frozen cell pellets were re-suspended in 1mL methanol and transferred to glass tubes. CHCl_3_ (3 mL) was added to each tube and sealed with foil to avoid contamination. The tubes were incubated for 5 min in a sonicator–water bath for cell disruption. MeOH/H_2_O (3:2) was added to the cell lysate and kept aside, to allow for phase separation. The final ratio of solvents MeOH/H_2_O/CHCl3 was 4:2:3. The 6mL of polar and 3 mL of non-polar phase were separated and kept on speed vacuum overnight. These vials were stored at −80 °C after sealing with parafilm for subsequent analysis.

### 4.3. Polar and Non-Polar General Metabolites Isolation

**Figure 5:**
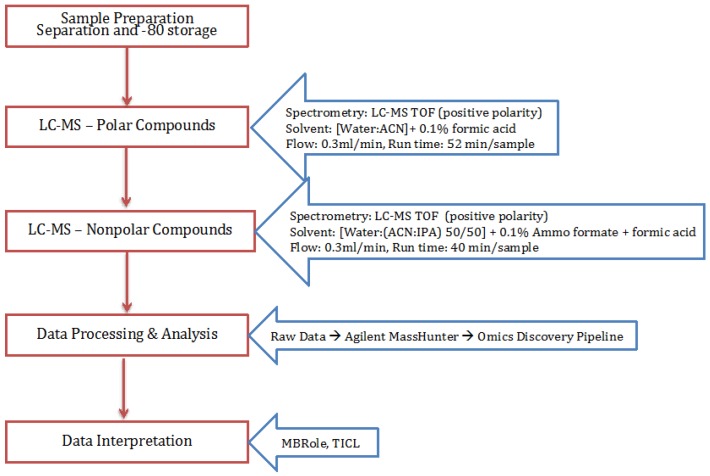
Flowchart for the method followed for the extraction, isolation, and detection of small molecules (general metabolites and lipids).

The processing of small molecules detection along with the instrumentation conditions are described in Figure 5.

### 4.4. Statistical Analysis Using Omics Discovery Pipeline

The raw data from the Agilent Mass Hunter software was fed to the Omics discovery pipeline developed by Purdue Bindley Biosciences Core Metabolite Care Lab Facility. This pipeline processed the data by performing deconvolution of peaks using XMASS. Preprocessing of *m*/*z*, intensity, and retention time data is fed to XALIGN to align the data from technical replicates, to reduce the possibility of instrumentation error. Further, *t*-tests were conducted to see significant difference from the blank runs, and eliminate the peaks due to solvents and spiked standards (mass of 121.050873 and 922.009798 respectively) [24].

### 4.5. Metabolite Identification and Pathway Association

**Figure 6:**
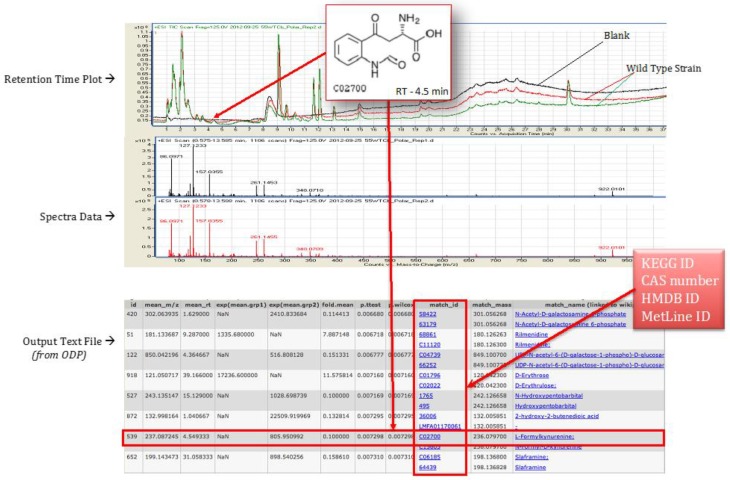
Illustration of retention time, spectra data peak, and sample output file obtained for *T. fusca* wild type strain grown on cellobiose growth media.

The polar and non-polar extracts were run as separate experiments, and the peak data was collected in the form of mzData files by the software Mass Hunter provided by Agilent. These mzData files were processed using Omics Discovery Pipeline (www.omicsDP.org) provided by Purdue [24]. An illustration of retention time plot, spectra data, and output of identified compounds is shown in Figure 6.

The identified set of metabolites was then mapped to *T. fusca* pathways using the precompiled compound list on KEGG [15,16] as a reference. This was done using MBRole [14], an online compilation of metabolites and pathways.

### 4.6. Metabolic Model and Metabolomics Integration

For a metabolic reconstruction, a flux distribution is a set of relative reaction rates that satisfy conservation of mass constraints. To derive a flux distribution that reflects the presence or absence of metabolites observed in metabolomics data, we adopt a simple modification of the standard flux balance analysis model. We find a flux distribution that maximizes the number of metabolites that are present in both the metabolomics data and our flux distribution. Presence of a metabolite in the metabolomics data is defined as being observed beyond a certain threshold in the metabolomics data. Presence of a metabolite in a flux distribution means that the sum of fluxes through reactions producing that metabolite exceeds a certain threshold.

Let R be the set of reactions and M be the set of metabolites that are in a draft metabolic reconstruction. Let $M^{\prime }$ be a subset of metabolites that are present in the metabolomics data. Let $S_{ij}$ be the stoichiometric coefficient for metabolite i in reaction j for i∈M,j∈R, with the convention that $S_{ij}$ is positive for reactions producing metabolite i, negative for reactions using metabolite i, and zero otherwise. Let $v_{j}$ be the flux through reaction j for j∈R. If a reaction is reversible, we represent the reaction as two irreversible reactions; because the columns in the stoichiometric matrix are linearly independent, only one direction will be selected in any basic optimal flux in our optimization model. In the following mixed integer program, $x_{i}$ equals 1 if metabolite i is present in both the metabolomic data and a flux distribution and 0 otherwise, for $i\in M^{\prime }$.

$$\begin{array}{l} \max\sum_{i\in M^{\prime }}x_{i},\\ s.t.\;\sum_{j\in R}S_{ij}v_{j}=0,i\in M,\\ \sum_{j\in R:i\in H_{j}}v_{j}\geq \varepsilon x_{i},i\in M^{\prime },\\ \ell_{j}\leq v_{j}\leq u_{j},j\in R,\\ x_{i}\in \{0,1\},i\in M^{\prime }. \end{array}$$

The objective function maximizes the number of metabolites present in both the metabolomics data and the flux distribution. The first set of constraints enforces conservation of mass. The next set of constraints require that if a metabolite is counted as present in the flux distribution, then the sum of fluxes producing that metabolite must be at least ε. For the experiments we conducted, we set ε=1.

## Figures and Tables

**Table 1:** Summary of compounds identified in the wild type strain of *Thermobifida fusca* when grown on cellobiose and Avicel media growth conditions.

Growth Condition	Total Detected Metabolites	Polar	Non-Polar	In Both
Cellobiose	667	618	64	15
Avicel	461	402	82	23

**Table 2:** Category distribution for metabolites detected in cellobiose (blue) and Avicel (red) growth condition. The total metabolites detected in cellobiose were 667 and in Avicel were 461.

Category	Total Relations		Number of Compounds		No Annotation *	
Pathways	72	71	191	129	476	332
Enzyme Interactions	299	213	71	45	595	416
Biological Roles	71	63	67	45	600	416
Chemical Groups	62	56	186	123	481	338
Other Interactions	95	74	31	25	636	436

* No annotation means, the MBRole found no connection of these compounds in any of the *T. fusca* pathways, Enzyme Interactions, Biological Roles, Chemical Groups or Other interactions as per KEGG’s annotation for *T. fusca*.

**Table 3:** Pathways associated with differentially detected compounds for growth on cellobiose vs. Avicel.

KEGG Pathway ID	Pathway	Detected	Compounds
Unique to Cellobiose			
tfu00230	Purine metabolism	15	C00362 C04376 C00360 C06196 C00130 C00035 C00242 C00655 C00330 C00262 C05515 C03794 C00212 C00048 C00385
tfu00240	Pyrimidine metabolism	8	C00364 C00099 C00112 C00015 C00380 C00055 C02376 C03997
tfu00270	Cysteine and methionine metabolism	7	C00109 C02989 C00041 C00073 C01005 C00021 C01180
tfu00330	Arginine and proline metabolism	6	C00791 C00555 C05931 C00062 C00437 C00048
tfu00450	Selenoamino acid metabolism	5	C05696 C05692 C00041 C05335 C05708
tfu00770	Pantothenate and CoA biosynthesis	4	C00099 C01088 C03492 C00864
tfu00740	Riboflavin metabolism	3	C05775 C00472 C00061
tfu00190	Oxidative phosphorylation	3	C00004 C00061 C00003
Unique to Avicel			
tfu00300	Lysine biosynthesis	5	C00047 C00322 C03340 C00049 C01251
tfu00330	Arginine and proline metabolism	5	C03440 C01043 C00049 C18174 C00327
tfu00906	Carotenoid biosynthesis	5	C16280 C08585 C08606 C15892 C08583
tfu00260	Glycine, serine and threonine metabolism	5	C03283 C06231 C00049 C00188 C00576
tfu02010	ABC transporters	4	C00049 C00047 C03557 C00188
tfu00970	Aminoacyl-tRNA biosynthesis	4	C00188 C00049 C00047 C00152
tfu00340	Histidine metabolism	3	C05575 C05131 C00049
tfu00250	Alanine, aspartate and glutamate metabolism	3	C00152 C00940 C00049
tfu00310	Lysine degradation	3	C00322 C00047 C00739
tfu00910	Nitrogen metabolism	2	C00152 C00049
tfu00641	3-Chloroacrylic acid degradation	2	C16348 C06613

**Table 4:** Compounds with significantly different concentrations as detected in two growth media conditions (green—cellobiose, red—Avicel).

KEGG Compound IDs	Description	Pathway ID	Name
Higher Concentration in Cellobiose			
C05763	(2E)-Hexadecenoyl-[acp]	tfu00061	Fatty acid biosynthesis
C03871	L-2-Amino-6-oxopimelate	tfu00300	Lysine biosynthesis
C12986	N2-Acetyl-L-aminoadipate	tfu00300	Lysine biosynthesis
C00624	N-Acetyl-L-glutamate	tfu00330	Arginine and proline metabolism
C03415	Succinyl-L-ornithine	tfu00330	Arginine and proline metabolism
C00513	CDP-glycerol	tfu00564	Glycerophospholipid metabolism
Higher Concentration in Avicel			
C00811	4-Coumarate	tfu00130	Ubiquinone & other terpenoid-quinone biosynthesis
C03955	N6-Acetyl-N6-hydroxy-L-lysine	tfu00310	Lysine degradation
C00811	4-Coumarate	tfu00360	Phenylalanine metabolism
C12836	2-Chloro-3-oxoadipate	tfu00361	gamma-Hexachlorocyclohexane degradation
C05699	L-Selenocystathionine	tfu00450	Selenoamino acid metabolism

**Table 5:** Distribution of Secondary Metabolites as detected in each of the growth conditions of cellobiose (Cb) and Avicel (Av).

	Both	Cb	Av	Total
Alkaloids	21	17	3	41
Alkaloids derived by amination reactions	6	1	1	8
Alkaloids derived from lysine	2	4	0	6
Alkaloids derived from nicotinic acid	2	2	0	4
Alkaloids derived from ornithine	7	1	1	9
Alkaloids derived from tryptophan and anthranilic acid	1	3	1	5
Alkaloids derived from tyrosine	3	3	0	6
Others	0	3	0	3
Amino acid related compounds	1	2	1	4
Betalains	1	2	1	4
Fatty acids related compounds	0	2	0	2
Fatty acids	0	2	0	2
Flavonoids	0	1	1	2
Flavonoids	0	1	1	2
Phenylpropanoids	3	4	1	8
Coumarins	1	2	1	4
Lignans	1	2	0	3
Monolignols	1	0	0	1
Polyketides	4	0	0	4
Others	2	0	0	2
Pyrones	2	0	0	2
Terpenoids	6	12	7	25
Carotenoids and apocarotenoids	0	0	3	3
Diterpenoids (C20)	0	4	1	5
Hemiterpenoids (C5)	1	0	0	1
Monoterpenoids (C10)	2	2	1	5
Sesquiterpenoids (C15)	3	3	1	7
Steroids	0	3	0	3
Triterpenoids (C30)	0	0	1	1
Others	2	0	0	2
Others	1	0	0	1
Tannins and galloyl derivatives	1	0	0	1
Total	37	38	13	88

**Table 6:** Computational predictions of fluxes through pathways for (**a**) amino acid metabolism and (**b**) carbohydrate metabolism using flux balance analysis (Flux-FBA) and after integrating metabolomic data for growth on cellobiose (Flux-WT-Cb).

**(a) Flux through Amino Acid Pathway before and after Metabolomics Data Integration**				
**Pathways**	**Flux−FBA**	**Flux−WT-Cb**	**ECs**	**Equation**
Alanine and Aspartate Metabolism	−1000.00	−7.55	4.3.2.1	[c]: C03406 <==> C00062 + C00122
	0.00	992.45	6.3.4.5	[c]: C00049 + C00002 + C00327 → C00020 + C03406 + C00080 + C00013
	973.09	2.27	2.6.1.1	[c]: C00026 + C00049 <==> C00025 + C00036
Arginine and Proline Metabolism	−1000.00	−7.55	3.5.3.6	[c]: C00062 + C00001 <==> C00327 + C01342
	978.81	6.61	1.5.1.2	[c]: C00003 + C00148 → C03912 + 2 C00080 + C00004
Glutamate Metabolism	19.12	3.75	6.3.1.2	[c]: C00002 + C00025 + C01342 → C00008 + C00064 + C00080 + C00009
	475.06	1000.00	1.4.1.2	[c]: C00025 + C00001 + C00003 → C00026 + C00080 + C00004 + C01342
Glycine and Serine Metabolism	8.41	1.65	1.8.1.4	[c]: C02972 + C00003 → C00080 + C02051 + C00004
	991.59	998.35	2.1.2.1	[c]: C00716 + C00101 → C00037 + C00001 + C00143
	1000.00	0.00	1.1.1.3	[c]: C00441 + C00080 + C00004 → C00263 + C00003
Glycine, Serine, and Threonine Metabolism	9.89	1.94	4.2.3.1	[c]: C00001 + C01102 → C00009 + C00188
Methionine Metabolism	0.31	0.06	2.5.1.6	[c]: C00002 + C00001 + C00073 → C00019 + C00009 + C00013
Phenylalanine, Tyrosine, and Tryptophan Biosynthesis	−994.44	0.00	4.1.1.48	[c]: C01302 <==> C03506 + C00011 + C00001
Threonine and Lysine Metabolism	−9.89	−1.94	1.2.1.11	[c]: C00441 + C00006 + C00009 <==> C03082 + C00080 + C00005
	9.89	1.94	2.7.2.4	[c]: C00049 + C00002 <==> C03082 + C00008
	9.89	1.94	2.7.1.39	[c]: C00002 + C00263 → C00008 + C00080 + C01102
	990.11	−1.94	1.1.1.3	[c]: C00263 + C00006 <==> C00441 + C00080 + C00005
Tyrosine, Tryptophan, and Phenylalanine Metabolism	994.44	0.00	4.1.1.48	[c]: C01302 + C00080 → C03506 + C00011 + C00001
Valine, Leucine, and Isoleucine Metabolism	1000.00	998.08	1.1.1.86	[c]: C04039 + C00006 <==> C06010 + C00080 + C00005
	1000.00	998.08	1.1.1.86	[c]: C06010 + C00080 + C00005 → C04039 + C00006
**(b) Flux Through Carbohydrate Pathway before and after Metabolomics Data Integration**				
**Pathways**	**Flux−FBA**	**Flux−WT-Cb**	**ECs**	**Equation**
Central Metabolism	961.51	0.00	2.7.9.1	[c]: C00002 + C00009 + C00022 → C00020 + C00080 + C00074 + C00013
	1000.00	663.00	1.1.1.49	[c]: f420-2 + C00668 → C01236 + f420-2h2
Citric Acid Cycle	−992.39	−6.06	4.2.1.2	[c]: C00122 + C00001 <==> C00711
	−973.09	−2.27	1.1.1.37	[c]: C00711 + C00003 <==> C00080 + C00004 + C00036
	−501.96	−331.89	6.2.1.5	[c]: C00002 + C00010 + C00042 <==> C00008 + C00009 + C00091
	501.96	331.89	1.2.4.2	[c]: C00026 + C00080 + C15972 <==> C00011 + C16254
	501.96	331.89	2.3.1.61	[c]: C00010 + C16254 → C00579 + C00091
Galactose Metabolism	−11.30	−2.22	2.7.7.9	[c]: C00103 + C00080 + C00075 <==> C00013 + C00029
	−11.30	−2.22	5.1.3.2	[c]: C00029 <==> C00052
Glycolysis/Gluconeogenesis	−1000.00	1000.00	5.3.1.9	[x]: g6p-a <==> C05345
	8.23	1.62	4.1.2.13	[c]: C00447 <==> C00111 + C00279
	8.23	1.62	2.7.1.11	[c]: C00002 + C05382 → C00008 + C00080 + C00447
	8.23	1.62	5.3.1.1	[c]: C00111 <==> C00118
	41.16	8.08	2.7.1.2	[c]: C00002 + C00267 → C00008 + C01172 + C00080
	41.16	8.08	5.3.1.9	[c]: C00668 <==> C05345
	961.51	0.00	2.7.1.40	[c]: C00008 + C00080 + C00074 → C00002 + C00022
	1000.00	−1000.00	5.3.1.9	[x] : C01172 <==> C05345
	1000.00	−1000.00	5.3.1.9	[x] : g6p-a <==> C01172
Glyoxylate and Dicarboxylate Metabolism	500.16	331.53	1.11.1.6	[c]: 2 C00027 → 2 C00001 + C00007
Pentose Phosphate Pathway	−41.16	328.92	1.1.1.49	[c]: C00668 + C00006 <==> C01236 + C00080 + C00005
	−32.93	−6.46	5.1.3.1	[c]: C00199 <==> C00231
	−24.69	−4.85	2.2.1.2	[c]: C00118 + C05382 <==> C00279 + C05345
	−16.46	−3.23	2.2.1.1	[c]: C03736 + C00231 <==> C00118 + C05382
	−16.46	−3.23	2.2.1.1	[c]: C00279 + C00231 <==> C05345 + C00118
	16.46	3.23	2.7.6.1	[c]: C00002 + C03736 <==> C00020 + C00080 + C00119
Starch and Sucrose Metabolism	−11.30	−2.22	2.4.1.18	[c]: C00718 <==> strch2_strch1
	−11.30	−2.22	2.4.1.1	[c]: strch2_strch1 + C00009 <==> C00718 + C00103

**Table 7:** Reactions that were found to be inactive after metabolomic data integration.

Pathways	Flux−FBA	Flux−WT-Cb	ECs	Equation
NAD Biosynthesis	−1000.00	**0.00**	2.4.2.11	[c]: C00080 + C11486 + C00119 <==> C01185 + C00013
Phenylalanine, Tyrosine, and Tryptophan Biosynthesis	−994.44	**0.00**	4.1.1.48	[c]: C01302 <==> C03506 + C00011 + C00001
Central Metabolism	961.51	**0.00**	2.7.9.1	[c]: C00002 + C00009 + C00022 → C00020 + C00080 + C00074 + C00013
Glycolysis/Gluconeogenesis	961.51	**0.00**	2.7.1.40	[c]: C00008 + C00080 + C00074 → C00002 + C00022
Nucleotide Metabolism	994.44	**0.00**	2.7.4.3	[c]: C00020 + C00536 <==> C00008 + C00013
Oxidative Phosphorylation	994.44	**0.00**	2.7.4.1	[c]: C00002 + C00013 → C00008 + C00536
Tyrosine, Tryptophan, and Phenylalanine Metabolism	994.44	**0.00**	4.1.1.48	[c]: C01302 + C00080 → C03506 + C00011 + C00001
NAD Biosynthesis	1000.00	**0.00**	2.4.2.11	[c]: C00080 + C11486 + C00119 → C01185 + C00013
Glycine and Serine Metabolism	1000.00	**0.00**	1.1.1.3	[c]: C00441 + C00080 + C00004 → C00263 + C00003

## Supplementary Materials

The following are available online at www.mdpi.com/2218-1989/7/4/57/s1, Figure S1: Cellobiose Media (a) Metabolic Process (b) Secondary Metabolites Process, Figure S2: Avicel Media (a) Metabolic Process (b) Secondary Metabolites Process, Table S1: Cellobiose Media Metabolites Annotation, Table S2: Avicel Media Metabolites Annotation, Table S3: Raw metabolomics data for growth on cellobiose and Avicel.

## References

[1] Lee J., Postmaster A., Soon H.P., Keast D., Carson K.C. (2012). Siderophore production by actinomycetes isolates from two soil sites in Western Australia. Biometals.

[2] Niraula N.P., Kim S.-H., Sohng J.K., Kim E.-S. (2010). Biotechnological doxorubicin production: Pathway and regulation engineering of strains for enhanced production. Appl. Microbiol. Biotechnol..

[3] Takahashi S., Toyoda A., Sekiyama Y., Takagi H., Nogawa T., Uramoto M., Suzuki R., Koshino H., Kumano T., Panthee S. (2011). Reveromycin A biosynthesis uses RevG and RevJ for stereospecific spiroacetal formation. Nat. Chem. Biol..

[4] Citron C.A., Gleitzmann J., Laurenzano G., Pukall R., Dickschat J.S. (2012). Terpenoids are widespread in actinomycetes: A correlation of secondary metabolism and genome data. Chembiochem.

[5] Cane D.E., Ikeda H. (2012). Exploration and mining of the bacterial terpenome. Acc. Chem. Res..

[6] Vanee N., Brooks J.P., Spicer V., Shamshurin D., Krokhin O., Wilkins J.A., Deng Y., Fong S.S. (2014). Proteomics-based metabolic modeling and characterization of the cellulolytic bacterium *Thermobifida fusca*. BMC Syst. Biol..

[7] Chen S., Wilson D.B. (2007). Proteomic and transcriptomic analysis of extracellular proteins and mRNA levels in *Thermobifida fusca* grown on cellobiose and glucose. J. Bacteriol..

[8] Deng Y., Fong S.S. (2010). Influence of culture aeration on the cellulase activity of *Thermobifida fusca*. Appl. Microbiol. Biotechnol..

[9] Deng Y., Fong S.S. (2010). Development and application of a PCR-targeted gene disruption method for studying CelR function in Thermobifida fusca. Appl. Environ. Microbiol..

[10] Deng Y., Fong S.S. (2011). Metabolic engineering of *Thermobifida fusca* for direct aerobic bioconversion of untreated lignocellulosic biomass to 1-propanol. Metab. Eng..

[11] Deng Y., Fong S.S. (2011). Laboratory evolution and multi-platform genome re-sequencing of the cellulolytic actinobacterium *Thermobifida fusca*. J. Biol. Chem..

[12] Spiridonov N.A., Wilson D.B. (1998). Regulation of biosynthesis of individual cellulases in *Thermomonospora fusca*. J. Bacteriol..

[13] Spiridonov N.A., Wilson D.B. (1999). Characterization and cloning of celR, a transcriptional regulator of cellulase genes from *Thermomonospora fusca*. J. Biol. Chem..

[14] Chagoyen M., Pazos F. (2011). MBRole: Enrichment analysis of metabolomic data. Bioinformatics.

[15] Kanehisa M., Goto S. (2000). KEGG: Kyoto encyclopedia of genes and genomes. Nucleic Acids Res..

[16] Kanehisa M., Goto S., Hattori M., Aoki-Kinoshita K.F., Itoh M., Kawashima S., Katayama T., Araki M., Hirakawa M. (2006). From genomics to chemical genomics: New developments in KEGG. Nucleic Acids Res..

[17] Ger M.-F., Rendon G., Tilson J.L., Jakobsson E. (2010). Domain-based identification and analysis of glutamate receptor ion channels and their relatives in prokaryotes. PLoS ONE.

[18] Adav S.S., Ng C.S., Sze S.K. (2011). iTRAQ-based quantitative proteomic analysis of *Thermobifida fusca* reveals metabolic pathways of cellulose utilization. J. Proteom..

[19] Guengerich F.P. (2008). Cytochrome p450 and chemical toxicology. Chem. Res. Toxicol..

[20] O’Loughlin S.N., Graham R.L.J., McMullan G., Ternan N.G. (2006). A role for carbon catabolite repression in the metabolism of phosphonoacetate by Agromyces fucosus Vs2. FEMS Microbiol. Lett..

[21] Kuzma J., Nemecek-Marshall M., Pollock W.H., Fall R. (1995). Bacteria produce the volatile hydrocarbon isoprene. Curr. Microbiol..

[22] Sharkey T.D., Wiberley A.E., Donohue A.R. (2008). Isoprene emission from plants: Why and how. Ann. Bot..

[23] Wang Y., Griffiths W.J. (2008). Capillary liquid chromatography combined with tandem mass spectrometry for the study of neurosteroids and oxysterols in brain. Neurochem. Int..

[24] Riley C.P., Gough E.S., He J., Jandhyala S.S., Kennedy B., Orcun S., Ouzzani M., Buck C., Roumani A.M., Zhang X. (2010). The proteome discovery pipeline—A data analysis pipeline for mass spectrometry-based differential proteomics discovery. Open Proteom. J..

