# Supplementary material for: Metabolic Profile of the Cellulolytic Industrial Actinomycete Thermobifida fusca
Source: Metabolites. 2017 Nov 11;7(4):57. doi: 10.3390/metabo7040057 (PMC5746737; doi:10.3390/metabo7040057)
Supplement: Supplementary file 1 [file metabolites-07-00057-s001.zip › metabolites-234040-proofreading-supplement/Supplementary Table 1 - Cellobiose Media Metabolites Annotation.pdf]

Pathway Distribution of Compounds Isolated from *T. fusca* grown on Cellobiose Media

KEGG Pathway ID	Pathway	Total	Detected	%	Compounds
**AMINO ACIDS METABOLISM**					
tfu00250	Alanine, aspartate and glutamate metabo	24	5	20.8%	C00041 C03794 C00169 C00025 C00122
					C05931 C00122 C00791 C00437 C03415 C00062 C00763 C00555
tfu00330	Arginine and proline metabolism	82	15	18.3%	C00048 C00169 C00624 C00148 C01250 C00025 C00431
tfu00300	Lysine biosynthesis	32	5	15.6%	C03871 C04421 C12986 C12987 C00449
tfu00400	Phenylalanine, tyrosine and tryptophan b	27	4	14.8%	C00296 C00166 C00108 C00078
tfu00270	Cysteine and methionine metabolism	56	8	14.3%	C02989 C00170 C00109 C00041 C01005 C00021 C01180 C00073
tfu00290	Valine, leucine and isoleucine biosynthesi	28	4	14.3%	C00407 C00123 C00109 C00183
tfu00360	Phenylalanine metabolism	46	6	13.0%	C00166 C02137 C05853 C00811 C00122 C02505
tfu00310	Lysine degradation	47	5	10.6%	C03656 C03955 C00408 C00449 C00431
tfu00260	Glycine, serine and threonine metabolism	49	5	10.2%	C00114 C00109 C00048 C01005 C00078
tfu00280	Valine, leucine and isoleucine degradatior	41	4	9.8%	C00164 C00407 C00183 C00123
tfu00380	Tryptophan metabolism	81	7	8.6%	C05659 C00078 C05660 C00108 C02700 C05837 C00643
tfu00350	Tyrosine metabolism	76	5	6.6%	C00164 C06199 C00483 C00122 C17938
tfu00340	Histidine metabolism	44	2	4.5%	C00025 C01152
**CARBOHYDRATES METABOLISM**					
tfu00640	Propanoate metabolism	36	8	22.2%	C02876 C00109 C00183 C00099 C00207 C05985 C00894 C00164
tfu00660	C5-Branched dibasic acid metabolism	32	4	12.5%	C00025 C02876 C00048 C00109
tfu00650	Butanoate metabolism	40	4	10.0%	C00122 C01384 C00025 C00164
tfu00020	Citrate cycle (TCA cycle)	20	2	10.0%	C00122 C05379
tfu00520	Amino sugar and nucleotide sugar metab	87	7	8.0%	C00140 C00128 C00043 C00203 C00270 C00029 C00645
tfu00052	Galactose metabolism	41	3	7.3%	C00029 C00116 C01286
tfu00630	Glyoxylate and dicarboxylate metabolism	44	3	6.8%	C00048 C02123 C00975
tfu00030	Pentose phosphate pathway	32	2	6.3%	C01236 C01151
tfu00620	Pyruvate metabolism	32	2	6.3%	C03981 C03248
tfu00500	Starch and sucrose metabolism	50	3	6.0%	C00029 C00689 C02591
tfu00053	Ascorbate and aldarate metabolism	47	1	2.1%	C00029
tfu00040	Pentose and glucuronate interconversion	53	1	1.9%	C00029
**BIOSYNTHSIS OF SECONDARY METABOLITES**					
tfu00900	Terpenoid backbone biosynthesis	33	2	6.1%	C16521 C00418
tfu00401	Novobiocin biosynthesis	37	2	5.4%	C12469 C00148
tfu00521	Streptomycin biosynthesis	24	1	4.2%	C01221
