# Supplementary material for: Metabolic Profile of the Cellulolytic Industrial Actinomycete Thermobifida fusca
Source: Metabolites. 2017 Nov 11;7(4):57. doi: 10.3390/metabo7040057 (PMC5746737; doi:10.3390/metabo7040057)
Supplement: Supplementary file 1 [file metabolites-07-00057-s001.zip › metabolites-234040-proofreading-supplement/Supplementary Table 2 - Avicel Media Metabolites Annotation.pdf]

Pathway Distribution of Compounds Isolated from *T. fusca* grown on Avicel Media

KEGG Pathway ID	Pathway	Total	Detected	%	Compounds
**AMINO ACIDS METABOLISM**					
tfu00300	Lysine biosynthesis	32	9	28.1%	C04421 C03340 C00449 C00049 C12986 C00047 C03871 C00322 C01251
tfu00250	Alanine, aspartate and glutamate metabolism	24	6	25.0%	C00169 C00152 C00049 C00940 C00025 C00122
tfu00330	Arginine and proline metabolism	82	14	17.1%	C03440 C00049 C00122 C00624 C01043 C00025 C18174 C00169 C00327 C00763 C00148 C01250 C00431 C03415
tfu00310	Lysine degradation	47	7	14.9%	C00739 C03656 C03955 C00431 C00322 C00047 C00449
tfu00290	Valine, leucine and isoleucine biosynthesis	28	4	14.3%	C00407 C00188 C00123 C00183
tfu00360	Phenylalanine metabolism	46	6	13.0%	C00811 C05853 C00122 C00166 C02137 C02505
tfu00260	Glycine, serine and threonine metabolism	49	6	12.2%	C00576 C00049 C03283 C00188 C00078 C06231
tfu00280	Valine, leucine and isoleucine degradation	41	4	9.8%	C00183 C00407 C00068 C00123
tfu00340	Histidine metabolism	44	4	9.1%	C00025 C00049 C05575 C05131
tfu00730	Thiamine metabolism	26	2	7.7%	C04327 C00068
tfu00400	Phenylalanine, tyrosine and tryptophan biosynthesis	27	2	7.4%	C00166 C00078
tfu00350	Tyrosine metabolism	76	4	5.3%	C00122 C00483 C17938 C01693
tfu00380	Tryptophan metabolism	81	3	3.7%	C00078 C00322 C05837
tfu00270	Cysteine and methionine metabolism	56	2	3.6%	C00049 C00170
tfu00450	Selenoamino acid metabolism	30	1	3.3%	C05699
**CARBOHYDRATE METABOLISM**					
tfu00640	Propanoate metabolism	36	5	13.9%	C00183 C00207 C02876 C00894 C05985
tfu00620	Pyruvate metabolism	32	4	12.5%	C03981 C00068 C01251 C03248
tfu00650	Butanoate metabolism	40	4	10.0%	C00025 C01384 C00122 C00068
tfu00020	Citrate cycle (TCA cycle)	20	2	10.0%	C00122 C00068
tfu00660	C5-Branched dibasic acid metabolism	32	2	6.3%	C00025 C02876
tfu00520	Amino sugar and nucleotide sugar metabolism	87	5	5.7%	C00140 C00645 C00029 C00043 C00203
tfu00052	Galactose metabolism	41	2	4.9%	C00029 C00116
tfu00040	Pentose and glucuronate interconversions	53	2	3.8%	C00029 C00085
tfu00010	Glycolysis / Gluconeogenesis	31	1	3.2%	C00068
tfu00030	Pentose phosphate pathway	32	1	3.1%	C01151
tfu00630	Glyoxylate and dicarboxylate metabolism	44	1	2.3%	C00975
tfu00053	Ascorbate and aldarate metabolism	47	1	2.1%	C00029
tfu00500	Starch and sucrose metabolism	50	1	2.0%	C00029
**BIOSYNTHSIS OF SECONDARY METABOLITES**					
tfu00906	Carotenoid biosynthesis	98	5	5.1%	C08583 C08585 C08606 C16280 C15892
tfu00900	Terpenoid backbone biosynthesis	33	1	3.0%	C16521
tfu00401	Novobiocin biosynthesis	37	2	5.4%	C12469 C00148

tfu01110	Biosynthesis of secondary metabolites	1038	37	3.6%	C00745 C00624 C00483 C00047 C03648 C05837 C01557 C00122 C00188 C00166 C00078 C12469 C17235 C00843 C00253 C00049 C03340 C00008 C00449 C01250 C00025 C16521 C05851 C17232 C10865 C00020 C00183 C00152 C00811 C03219 C06185 C09910 C00148 C00407 C00327 C00123 C00029
