# Supplementary figures and images for: Metabolic Profile of the Cellulolytic Industrial Actinomycete Thermobifida fusca
Source: Metabolites. 2017 Nov 11;7(4):57. doi: 10.3390/metabo7040057 (PMC5746737; doi:10.3390/metabo7040057)

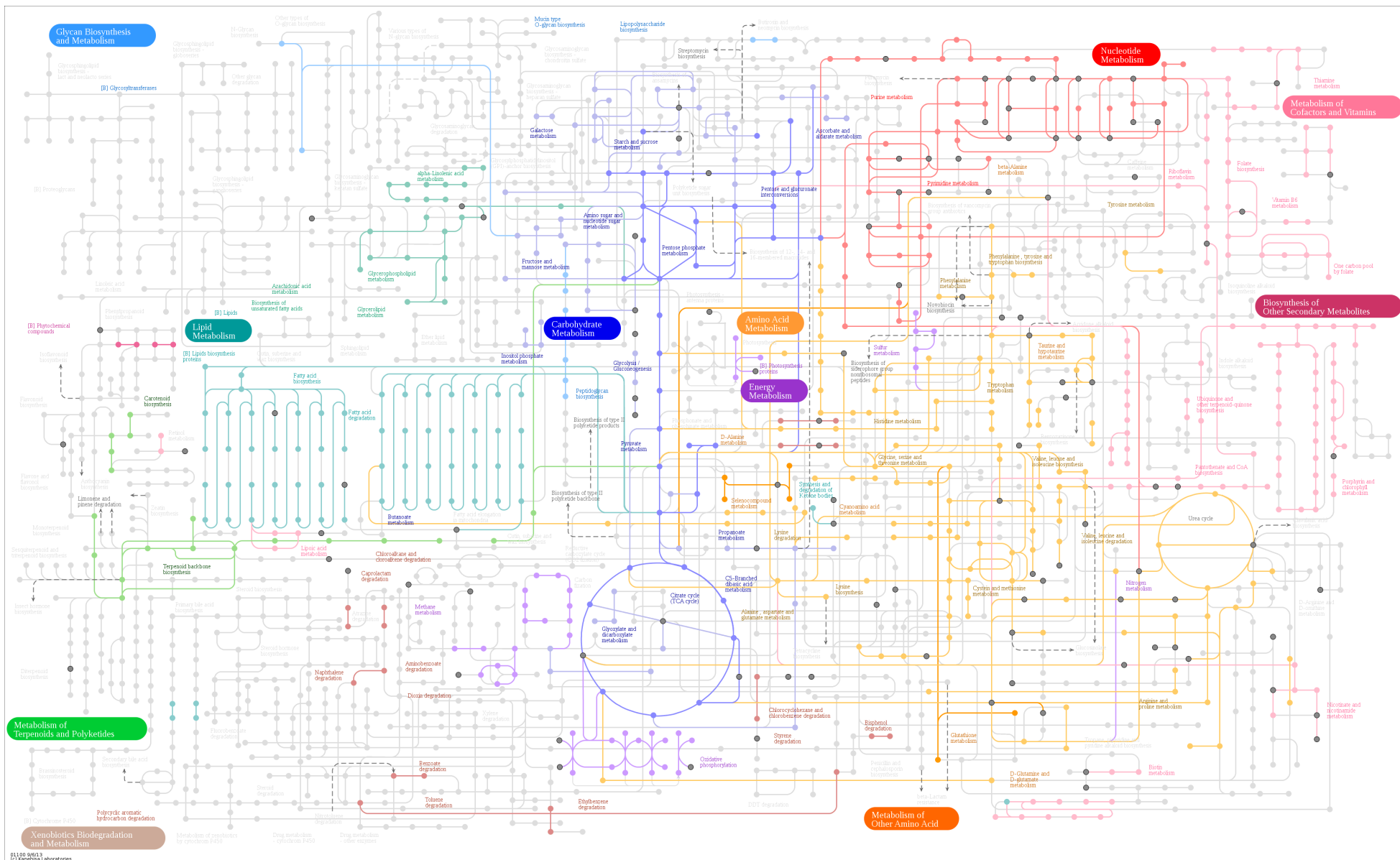

Supplement: Supplementary file 1 [file metabolites-07-00057-s001.zip › metabolites-234040-proofreading-supplement/Supplementary Figure 1a - Cellobiose Media_MetabolicProcess.pdf]

Metabolism of  
Terpenoids and Polyketides

Biosynthesis of  
Other Secondary Metabolites

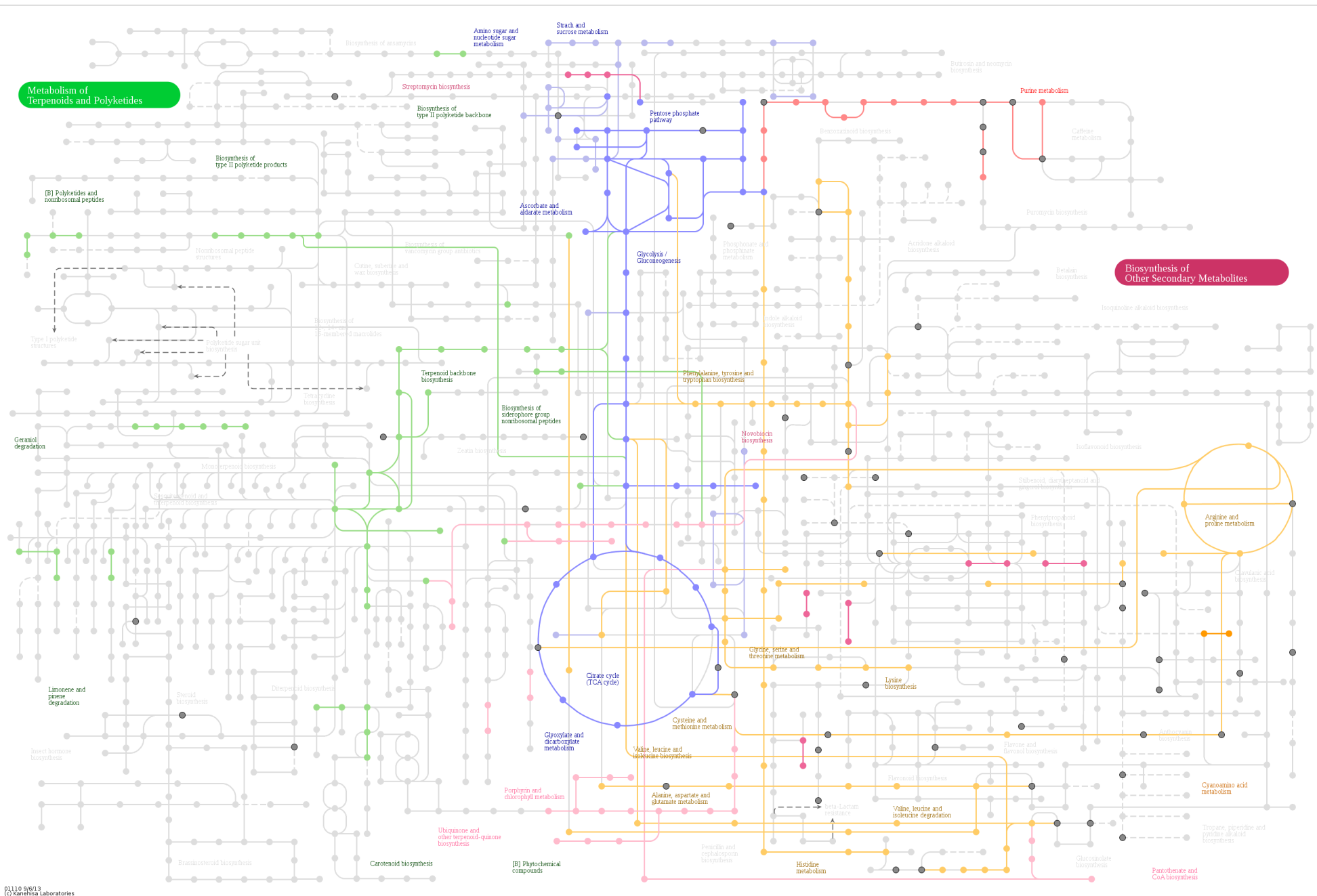

Supplement: Supplementary file 1 [file metabolites-07-00057-s001.zip › metabolites-234040-proofreading-supplement/Supplementary Figure 1b - Cellobiose Media_Secondary Metabolites Process.pdf]

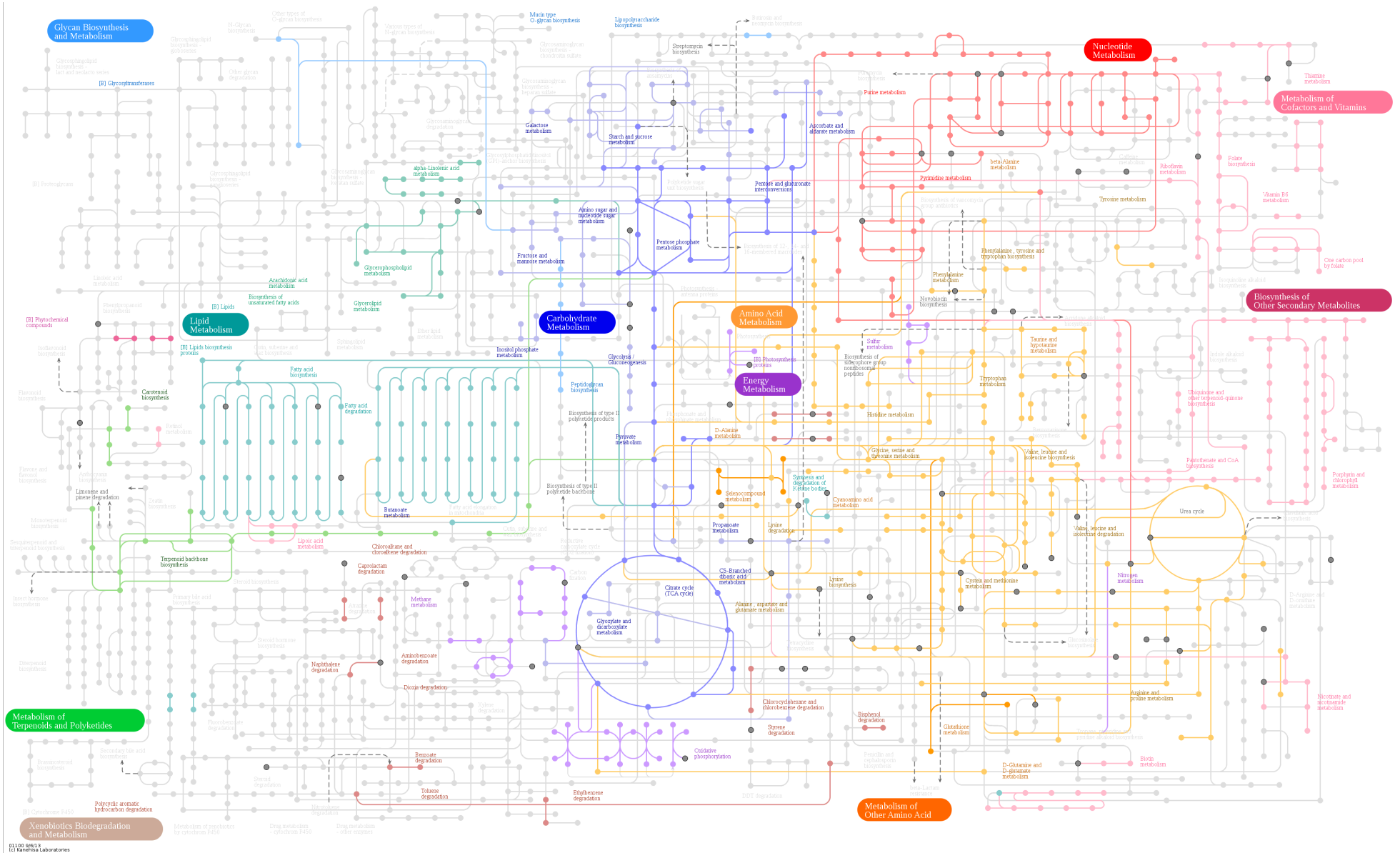

Supplement: Supplementary file 1 [file metabolites-07-00057-s001.zip › metabolites-234040-proofreading-supplement/Supplementary Figure 2a - Avicel Media_MetabolicProcess.pdf]

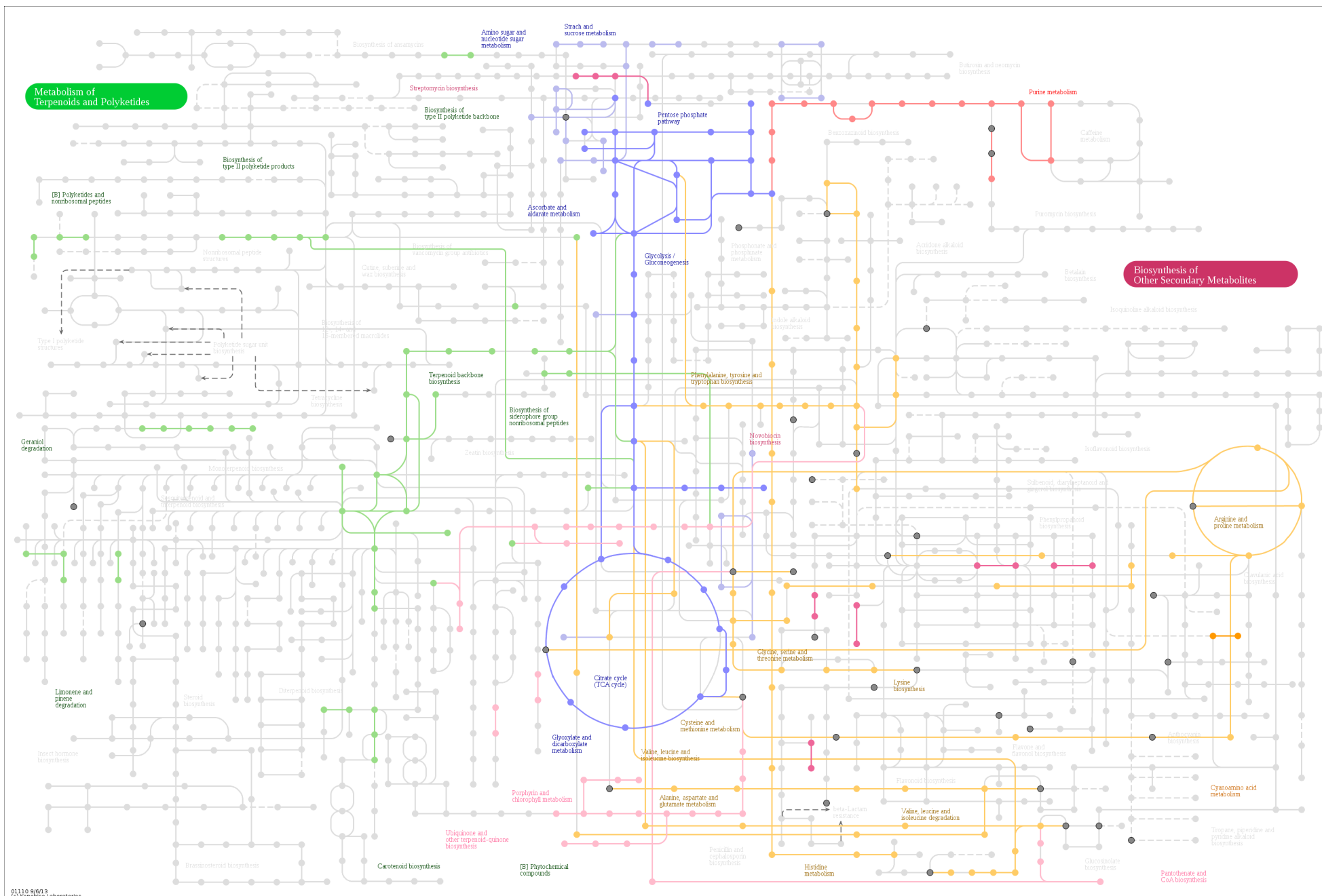

Supplement: Supplementary file 1 [file metabolites-07-00057-s001.zip › metabolites-234040-proofreading-supplement/Supplementary Figure 2b - Avicel Media_Secondary Metabolites Process.pdf]
